# New Horizons in Probiotics: Unraveling the Potential of Edible Microbial Polysaccharides through In Vitro Digestion Models

**DOI:** 10.3390/foods13050713

**Published:** 2024-02-26

**Authors:** Yuying Wang, Shengyong Zhu, Tiantian Zhang, Minjie Gao, Xiaobei Zhan

**Affiliations:** Key Laboratory of Carbohydrate Chemistry and Biotechnology, Ministry of Education, School of Biotechnology, Jiangnan University, Wuxi 214122, China; yuyingefficiency@gmail.com (Y.W.);

**Keywords:** in vitro digestion models, microbial polysaccharides, xanthan gum, gellan gum, probiotic potential

## Abstract

In vitro digestion models, as innovative assessment tools, possess advantages such as speed, high throughput, low cost, and high repeatability. They have been widely applied to the investigation of food digestion behavior and its potential impact on health. In recent years, research on edible polysaccharides in the field of intestinal health has been increasing. However, there is still a lack of systematic reviews on the application of microbial-derived edible polysaccharides in in vitro intestinal models. This review thoroughly discusses the limitations and challenges of static and dynamic in vitro digestion experiments, while providing an in-depth introduction to several typical in vitro digestion models. In light of this, we focus on the degradability of microbial polysaccharides and oligosaccharides, with a particular emphasis on edible microbial polysaccharides typically utilized in the food industry, such as xanthan gum and gellan gum, and their potential impacts on intestinal health. Through this review, a more comprehensive understanding of the latest developments in microbial polysaccharides, regarding probiotic delivery, immobilization, and probiotic potential, is expected, thus providing an expanded and deepened perspective for their application in functional foods.

## 1. Introduction

In recent decades, extensive and in-depth research has been conducted on the benefits of oligosaccharides and polysaccharides on the intestinal microbiota and microenvironment. Among them, inulin and oligofructose, as representatives of great interest, have demonstrated selective regulation of the intestinal microbiota, both in vivo and in vitro [1,2,3]. In addition to carbohydrates derived from plants, researchers have progressively directed their attention towards microbial exopolysaccharides and their degradation products as potential prebiotics in recent years. Microbial extracellular polysaccharides are produced through the fermentation of various microorganisms, offering the advantage of low-cost mass production. These polysaccharides are combined with probiotics in the form of capsules, hydrogels, nanoparticles, microspheres, and matrix tablets to form synbiotics, which are widely used in the food and pharmaceutical industries [4,5]. Some physiologically functional polysaccharides are commonly employed to regulate intestinal health and have extensive applications in the food industry. Gellan gum (GG) is an anionic polysaccharide characterized by strong gelling ability, acid resistance, and good thermal stability [6,7,8]. When combined with calcium ions, GG forms a gel network structure, and it is widely applied in the controlled release of drugs and bioactive substances [9]. Chitosan is the only cationic polysaccharide in nature, and protonated chitosan can form a complex with anionic polysaccharides, like GG, through electrostatic interactions, thus enhancing the strength of the GG microgel system [10]. In addition to the above reports, the consumption of prebiotic oligosaccharides has been proven to positively affect the absorption of calcium, magnesium, and iron, as well as to combat diseases such as cardiovascular disease, cancer, obesity, and type II diabetes [11,12]. Similarly, xanthan gum (XG) has been proven to reduce metabolic pathway disturbances in the intestines, and it serves as a crucial targeted delivery material for nutrients and active substances, exhibiting pH responsiveness and controllable release advantages [13]. Chito-oligosaccharides improve intestinal immunity in piglets challenged by enterotoxigenic Escherichia coli by partially modulating the microbial community and immune stress response through specific immune signals. Additionally, chito-oligosaccharides enhance villus length and improve the morphological structure of the ileum [14]. Supplementation of lactulose oligosaccharides can reverse nutritional deficiencies in the intestinal microbiota of rats suffering from alcohol withdrawal syndrome and restore intestinal permeability [15]. Degradation products of microbial polysaccharides, such as xylose and lactulose oligosaccharides, have been found to increase the relative abundance of bifidobacteria and the production of short-chain fatty acids in animal and in vitro fermentation studies [16,17]. Konjac glucomannan has been demonstrated to regulate the intestinal microbiota and reduce obesity rates in mice [18]. Furthermore, some microbial polysaccharides, such as fructo-oligosaccharides, galacto-oligosaccharides, and ꞵ-glucans, have been confirmed to regulate the human intestinal microbiota and influence growth performance and immunity in rat models [19].

Given the growing interest in healthy eating and the increasing prevalence of food-related diseases such as type II diabetes, cardiovascular diseases, and food allergies, a thorough understanding of the impacts of food structure and components on human health becomes crucial. Therefore, in vitro digestion models, as promising assessment tools, can track the digestion behavior of a food and estimate its impact on health. Compared to in vivo methods, in vitro models have the advantages of being faster, as well as having higher throughput, lower cost, and greater repeatability. These models are not hindered by ethical constraints, so, over the past few decades, research on microbial polysaccharides and their impacts on intestinal health has gradually increased. However, there has not been a systematic review of the application of microbial-derived edible polysaccharides in in vitro intestinal models. Existing studies indicate that microbial-derived polysaccharides may influence host health by regulating the structure and metabolism of the intestinal microbiota, but the relevant mechanisms still require further exploration [11,12]. Of particular interest are XG and GG, two representative microbial-derived colloids that have potential prebiotic effects as edible polysaccharides. By delving into the behaviors of these colloids during the simulation of human intestinal digestion, the aim is to provide a theoretical foundation for further research in this field and guide the development of edible polysaccharide colloids with potential prebiotic effects.

## 2. In Vitro Digestion Models

**Figure 1:**
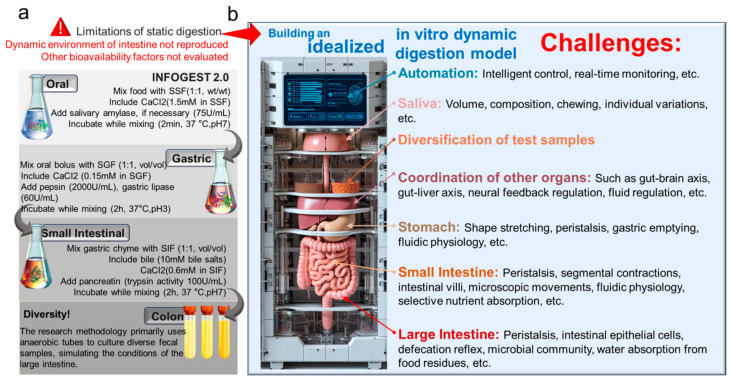
(**a**) International Consensus: Process of INFOGEST 2.0 in vitro static digestion standard (oral, gastric, small intestinal) and anaerobic cultivation of fecal microbiota [21]; (**b**) numerous challenges faced by idealized in vitro dynamic digestion.

The evolving food industry has accentuated the significance of in vitro simulation for studying the human digestive system. This simulation method is broadly classified into static and dynamic forms. While static simulation offers a quick and flexible solution, it falls short in replicating mechanical and biochemical changes like gastric fluid secretion, pH variations, and enzyme activity. Notably, it struggles to mirror dynamic fluid and peristaltic movements, complicating the correlation with in vivo conditions. Additionally, static simulation inadequately supports the survival and proliferation of microbial communities, thus hindering accurate representation of complex interactions. When considering the impacts of individual differences on the digestive process, static simulation methods exhibit diverse variations. Reports indicate that, in studies using static simulation for gastric digestion, even up to four different protein enzyme concentrations have been observed, and, in starch digestion studies, as many as 36 static methods have been reported [20]. To standardize static simulation research methods, Europe has established a standardized experimental protocol for static oral, gastric, and intestinal simulations with good reproducibility, as depicted in Figure 1a [21]. Static simulation has been widely employed in the study of glucose release from carbohydrate-containing foods, such as rice, bread, and oats [22]. It is also used to estimate the glycemic indices of foods and assess their rapid digestion (within 20 min), slow digestion (within 120 min), and resistant starch contents (not hydrolyzed after 120 min) [23,24]. While static digestion methods have some apparent drawbacks, they remain the most commonly used in vitro digestion approaches. This is because dynamic digestion methods face a multitude of diverse and complex challenges, with their ultimate goal being to simulate the physiological environment of the human body as closely as possible during the simulation process (Figure 1b). Gastrointestinal dynamic simulation effectively models food digestion, which is preferred for studying fluid dynamics and digestion rates. In vitro human gastrointestinal simulation systems replicate physiological functions, providing a simple, convenient, safe, and ethical alternative for research that is widely applied in assessing food safety, gastrointestinal metabolic dynamics, and nutritional substance bioavailability [25,26]. Currently, in vitro intestinal digestion models are broadly categorized into static single-chamber, dynamic single-chamber, dynamic double-chamber, and dynamic multi-chamber models [25].

These models can be either discontinuous or continuous [27]. Discontinuous models are simpler, comprising a closed bioreactor typically controlling temperature, oxygen, and pH values. Continuous models more accurately reflect in vivo conditions, including nutrient inflow and waste outflow. Numerous available continuous and discontinuous validation models are often used in microbial community studies, such as the EnteroMix, PolyFermS, CoMiniGut, TIM-1 and TIM-2, SHIME, or SIMGI models [28,29,30].The fourth-generation Dynamic Human Stomach Intestine (DHSI-IV) and the bionic gastrointestinal reactor (BGR) are also utilized to simulate the impact of food on the survival characteristics of probiotics in the digestive process [31,32]. Currently, various dynamic simulation devices have been developed and applied abroad; several typical intestinal reactors will be highlighted in the following sections.

### 2.1. The Earliest—SHIME

Molly, K. et al. first developed the computer-controlled multi-compartment dynamic digestion model in 1993, known as the SHIME [33]. The model is composed of five compartments that replicate the physiological conditions of both the upper and lower gastrointestinal tracts, specifically encompassing the stomach, small intestine, ascending colon, transverse colon, and descending colon. Each reactor is constituted by five double-walled glass vessels, meticulously maintained at a temperature of 37 °C, and interlinked by peristaltic pumps. The testing duration can vary, spanning from 24 to 76 h. To uphold anaerobic conditions within the lower gastrointestinal tract, a daily application of N_2_, or an N_2_/CO_2_ mixture, is employed to purge the upper space of the relevant compartments. The initial two reactors, emulating the stomach and small intestine, function based on the fill-and-draw principle. This involves the systematic addition of a quantitative blend of nutrient medium, pancreatic enzymes, and bile into the simulator (Supplementary Figure S1a). Conversely, the three subsequent colon compartments experience continuous stirring to maintain consistent volumes of 0.5 L, 0.8 L, and 0.6 L, respectively. The pH values within these compartments are carefully regulated within the ranges of 5.6~5.9, 6.1~6.4, and 6.6~6.9 to mimic the physiological conditions of the human colon. The total residence time for the last three compartments simulating the large intestine is 72 h [34]. A notable feature of this model is its capability to investigate the biochemical processes of chemical substances throughout the entire digestive system, from entry into the stomach to excretion, and to delve into the metabolic behavior of intestinal microbiota towards these substances [35]. The M-SHIME, building upon the SHIME, introduces microorganisms covered with mucin and a mucosal environment, providing a more precise simulation of the human intestinal tract, including the protective mucous layer [36]. This model not only simulates suspended microorganisms, but also replicates surface-adhered intestinal microbiota and mucin-degrading communities. Consequently, the dynamic intestinal model of the M-SHIME allows for the dynamic assessment of various colon regions, maintaining differences among individual humans, and exploring the interactions between microbiota and hosts, which is particularly relevant in the field of inflammatory bowel diseases (Supplementary Figure S1b).

Currently, the SHIME is widely utilized for functional evaluations related to food, prebiotics, toxic substances, and the study of how the digestive environment influences microbial diversity and metabolic activity. One representative study involves the evaluation of plant polysaccharides using the SHIME, wherein soybean polysaccharides were found to reduce the ratio of *Firmicutes* to *Bacteroidetes* in the SHIME system, promoting the growth of probiotics such as *Bifidobacterium* and *Lactobacillus*, and enhancing the inhibitory capacity against pathogenic bacteria. Furthermore, the production of short-chain fatty acids (SCFAs), metabolic products of the intestinal microbiota, significantly increased, suggesting that soybean polysaccharides can be used in the production of health-promoting foods for intestinal health [37,38]. Another study found that Ganoderma polysaccharides are utilized as a source of energy in the intestinal microbiota, simultaneously promoting the production of short-chain fatty acids, such as acetic acid, propionic acid, and butyric acid. This presents a novel form of prebiotic with potential benefits for human health [39].

### 2.2. Widely Used—TIM

The TIM is a dynamic and flexible simulation device for the human gastrointestinal tract, developed by Minekus, M. [29]. It uses smooth and elastic silicone rubber to mimic the stomach, duodenum, jejunum, ileum, and colon. Encased in a transparent glass jacket filled with water, it simulates the peristaltic, contraction, and relaxation movements of the gastrointestinal wall by changing water pressure. Additionally, it is computer-controlled to regulate the main physiological parameters and digestive secretions in the gastrointestinal tract (Supplementary Figure S2a). This model effectively simulates the physical, chemical, and physiological processes of probiotics or fiber in the gastrointestinal tract [40,41,42]. The latest development from TNO, the TIM-2, is a computer-controlled bioreactor system designed to simulate the proximal part of the human colon, as described by Venema, K. [28]. The TIM-2 system, an extension of the TIM-1, has been effectively utilized in various applications. This dynamic computer-controlled model accurately simulates the proximal portion of the human colon. Its core features include interconnected glass containers with flexible walls, through which water is periodically pumped to replicate intestinal peristalsis. A dialysis system ensures the removal of water and fermentation by-products to maintain physiological conditions. Food, mimicking the composition of ileal efflux medium, is introduced via an inlet system. Operating under anaerobic conditions at a constant 37 °C, the system maintains a stable pH of 5.8 via sensor control. A simplified illustration of the TIM-2 is depicted in Supplementary Figure S2b. Upon introduction of human microbiota, the system undergoes a 16 h lag phase, followed by nutrient depletion before testing initiation.

### 2.3. Unique—PolyFermS

**Figure 2:**
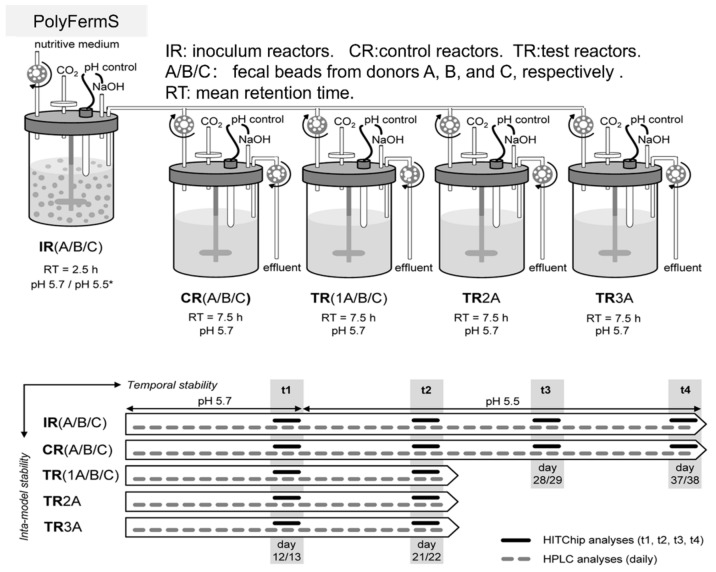
Schematic representation of the PolyFermS platform [45]. * The pH was automatically controlled at 5.7 by adding 2.5 N NaOH during the first 12 days and was decreased thereafter to pH 5.5 in IR after 13 days to account for initial metabolic imbalances and account for a likely lower pH in this section of the colon with high microbial activity.

The PolyFermS platform stands out as the pioneering fermentation model featuring immobilized fecal microbiota, and it is widely employed to explore the ecology and metabolism of the intestinal microbiota. It encapsulates fresh fecal microbiota in gel beads, facilitating exponential growth to high density before releasing bacteria into the fermentation medium (Figure 2). Focused on the colonic microbiota, the PolyFermS platform utilizes immobilized fecal microbiota continuously inoculated into parallel secondary reactors. The model employs fecal samples from healthy individuals, immobilizing the microbiota in gel beads composed of carrageenan, XG, and sodium citrate, which sets the PolyFermS platform apart as a distinctive entity among various intestinal models [43,44,45]. Compared to other intestinal fermentation models, this model has the capability to cultivate complex colonic microbial communities in a parallel, stable, and reproducible manner across multiple reactors. It enables the simultaneous investigation of various factors (such as environmental parameters, dietary compounds, drugs, and added microorganisms) and their effects with appropriate controls. The model is extendable to donors of infants, elderly individuals, or those with obesity. It has the capability to stably cultivate complex colonic microbial communities from fecal donors. This allows for comparisons between controls and different treatment outcomes within the same microbial community, making it highly suitable for investigating the mechanisms of action of various prebiotics and bacterial metabolites [46,47,48,49]. Recently, a significant amount of research has been dedicated to exploring mechanisms related to the intestine using the PolyFermS platform. Poeker, S.A. et al. innovatively developed a continuous fermentation model utilizing the PolyFermS platform, specifically tailored for the murine cecum and inoculated with immobilized cecal microbiota [50]. The ingestion of probiotic bacteria may result in their transient colonization in the human intestinal tract, potentially interacting with the commensal microbiota. Fehlbaum, S. et al. harnessed the PolyFermS platform to explore interactions involving the candidate probiotic strain *Lactobacillus paracasei* CNCM I-1518 with the colonic microbiota of healthy elderly individuals. The study also examined interactions with a gut pathogen prevalent in the elderly population, employing 16S rRNA gene amplicon sequencing and metatranscriptomics [51]. The PolyFermS platform generates planktonic and sessile “artificial” colonic microbiota, thus offering a controllable and reproducible alternative to fecal transplantation for treating gastrointestinal disorders. In a study by Bircher, L. et al., the planktonic and sessile microbiota produced in two PolyFermS platforms, inoculated with immobilized fecal microbiota, were characterized. Tolerance levels to frozen storage (−80 °C) and freeze-dried storage (4 °C) were comparatively tested for 9 months, simulating preservation strategies for therapeutic applications [52]. Naimi, S. et al. focused on evaluating the inhibitory activity of Microcin J25 against the *Salmonella Newport* subspecies [53]. Simultaneously, Isenring, J. et al. aimed to establish the PolyFermS platform as a tool for adaptive evolutionary engineering. The data suggested that the PolyFermS platform is a technology suitable for generating adapted mutants under colonic conditions [46]. Further research led to the generation of adapted mutants of *Lactiplantibacillus plantarum* NZ3400, a strain derived from *Lactobacillus plantarum* WCFS1. These mutants were adapted to the conditions of the adult colon in the PolyFermS platform [54]. Gosciniak, A. et al. highlight their importance in contributing to disease understanding and present in vitro models enabling the study of microbiota under near-natural conditions [55]. In the study by Naimi, S. et al., the impact of Microcin J25 on the composition and metabolic activity of swine colonic microbiota is assessed. The PolyFermS platform was employed, utilizing a modified Macfarlane medium to simulate the porcine proximal colon [56].

### 2.4. Emerging—BGR

**Figure 3:**
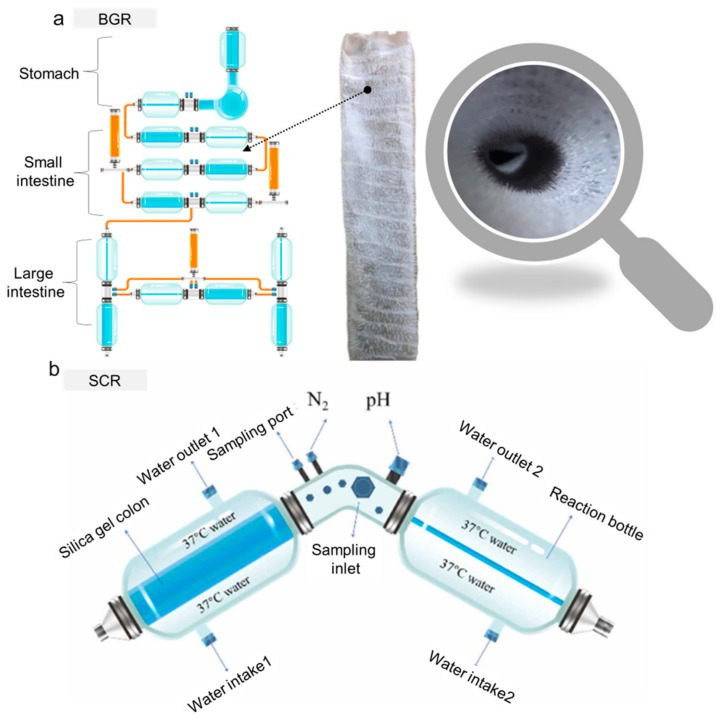
(**a**) Schematic representation of the BGR [32]; (**b**) schematic representation of the SCR [57].

Li, Z.T. et al. successfully developed a novel dynamic digestion model reactor in 2020, named the BGR, which is capable of simulating gastrointestinal functions, with a maximum of nine chambers [32]. These chambers simulate the fundus, body, antrum, duodenum, jejunum, ileum, Transverse colon, ascending colon, descending colon. These compartments are easy to disassemble, convenient for sterilization, and can be used independently or in series. To enhance the efficiency and reliability of experiments, the research team developed both offline control systems and online cloud platform control systems, enabling precise control of peristaltic frequency, secretion rate, real-time pH curves, historical data export, and operation status alarms. Additionally, researchers prepared smooth silicone stomachs and intestines, as well as wrinkled silicone stomachs and villous intestines. The folded inner walls increased wrinkles and villi, enlarging the surface area inside the intestine, altering the rheological forces of chyme, promoting food fragmentation, and facilitating microbial colonization in the intestine (Figure 3a). This reactor exhibited good mixing effects for both Newtonian and non-Newtonian fluids, simulating the peristaltic contraction and powerful contraction stages of the stomach. It possesses the ability to fragment solid food and dynamically adjust the pH during the digestion process. Building upon the BGR, Li, Z.T. et al. further developed an in vitro simulation colon reactor (SCR) in 2023 [57]. The SCR exhibited good mixing effects, improved both mass transfer and heat transfer, and simulated both low- and high-frequency peristaltic movements, thus resembling the human body (Figure 3b). This reactor could simulate the absorption of metabolites, allowing for microbial growth without inhibition. It resisted contamination, even after prolonged operation, ensuring experimental accuracy. The reactor also possessed excellent acid–base balance regulation capabilities, ensuring normal microbial growth. Most importantly, after colonization in the reactor, the similarity rate of fecal microbial communities to the initial fecal microbial communities exceeded 85%, demonstrating a satisfactory colonization effect. These two reactors are among the more complete functional gastrointestinal reactors developed in recent years, having successfully achieved applications such as microbial colonization, digestion monitoring, and functional evaluation. Building on the foundation of the BGR, Li, Z.T. et al. conducted a study on the growth and metabolism of *Akkermansia muciniphila* (*A. muciniphila*), revealing that dynamic cultivation yielded higher biomass compared to static cultivation [58]. They optimized the cultivation medium for *A. muciniphila*, finding that the human mucin protein medium was more suitable for the growth and metabolism of *A. muciniphila* compared to the pig mucin protein medium. The study unveiled that the nutritional components and cultivation conditions directly affected the biomass, outer membrane protein concentration, thickness, and cell diameter of *A. muciniphila*. The BGR is also widely applied in the digestion detection of sugar substances in food. By studying various processing methods for rice high in resistant starch, it was discovered that rice cakes had the highest content of resistant starch, while rice flours had the lowest. The digestion rates of starch in the foods followed a first-order two-phase equation. Compared to inulin, rice cakes had a slower fermentation rate, providing energy to the distal colon and promoting the growth of *Prevotellaceae*, which contributes to short-chain fatty acid synthesis, and the anti-inflammatory *Faecalibacterium*. The growth inhibition of imbalance indicators *Proteobacteria* and *Megamonas* in the intestinal microbiota was observed, revealing the mechanism by which rice high in resistant starch improves the structure and function of the intestinal microbiota [59,60]. Yu, D. et al. employed the BGR for an in vitro digestion simulation investigating the impact of steamed buns made from refined flour, 1:1 mixed flour, and whole wheat flour on the intestinal microbiota. In comparison to the buns made with refined flour and mixed flour, the addition of 0.5% whole wheat flour resulted in a significant change in butyric acid and short-chain fatty acid contents (*p* < 0.05). Whole wheat flour promoted the growth of beneficial microbial communities, such as *Megamonas* and *Subdoligranulum*. Furthermore, the addition of 0.5%, 1.0%, and 1.5% whole wheat flour led to a significant increase in acetic acid and short-chain fatty acids. Whole wheat flour also stimulated the proliferation of *Bifidobacterium*, *Lactobacillus*, and *Bacteroides*, while inhibiting the growth of pathogenic microbial communities [61]. These studies provide insights for the development of new functional starch-derived food products. In the study of dietary fatty acid regulation on intestinal gas distribution, the BGR digestion experiments showed that the gas components produced by fecal microbiota using dietary fatty acids are mainly CO_2_, H_2_, H_2_S, and VOC_S_, stimulating an increase in H_2_S and VOC_S_ concentrations in the intestines [60]. This provides mechanistic evidence for the increased incidence of intestinal diseases caused by high-fat diets and offers a basis for designing dietary strategies to reduce intestinal H_2_S and VOC_S_ concentrations. It is worth noting that the BGR is currently widely used for monitoring the digestion of microbial polysaccharides.

## 3. Factors Influencing the Impacts of Microbial Polysaccharides and Oligosaccharides on the Intestine

**Figure 4:**
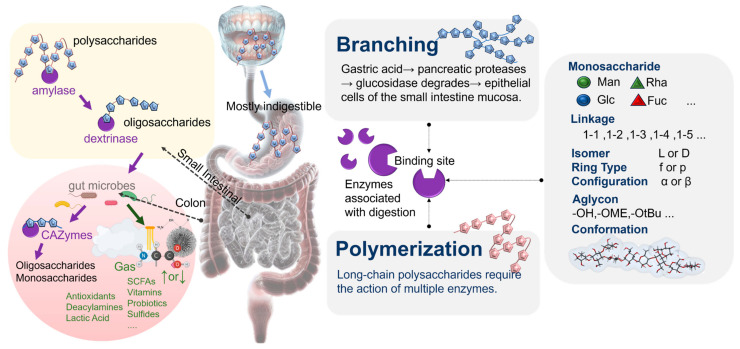
The potential modes of microbial polysaccharide digestion and the structural and physicochemical factors influencing the degradability of microbial polysaccharides during digestion.

Most microbial polysaccharides, due to their complex structures, can maintain relative stability in their structures through the processes of oral and gastric digestion. In contrast, in the small intestine, microbial polysaccharides with high polymerization and branching often undergo partial degradation. During colonic fermentation, certain unique chemical structures of microbial polysaccharides can selectively promote the proliferation of intestinal microbiota and induce the secretion of specific glycosidases, leading to the degradation of polysaccharides with specific structural types. This process is accompanied by the production of metabolites by the intestinal microbiota. Upon reaching the colon, these oligosaccharides are primarily metabolized by *Bifidobacterium*, *Lactobacillus*, and other symbiotic bacteria containing various carbohydrate metabolism-related enzymes. Upon ingestion, oligosaccharides are first recognized and hydrolyzed into low-degree oligosaccharides by modular glycosyl hydrolases on the bacterial membrane. Subsequently, these oligosaccharides enter the cell through membrane protein transport and are enzymatically broken down into disaccharides and monosaccharides (Figure 4) [62]. For instance, oligofructose is first absorbed by *Lactobacillus acidophilus* through the ABC (ATP-binding cassette) transport system, degraded into β-glucosides, and then enzymatically decomposed into fructose and glucose through the actions of fructose glycosidase and β-furan glycosidase, which are subsequently utilized by the cell [63,64]. Andersen et al. utilized differential transcriptomics and functional genomics to elucidate the uptake and catabolism mechanisms of various potential prebiotic compounds by *Lactobacillus acidophilus* NCFM. The study revealed that oligosaccharides induced a series of gene expressions, including the phosphoenolpyruvate-dependent sugar phosphotransferase system (PTS), galactoside pentose hexuronide permease, and ABC transporters. Specifically, disaccharides and trisaccharides such as cellobiose, isomaltose, isomaltulose, panose, and gentiobiose predominantly upregulated the PTS system, while glucose, polydextrose, and low-degree oligosaccharides upregulated ABC transporters. Additionally, lactitol and galacto-oligosaccharides induced specific GPH transporters [65]. For intestinal bacteria, the abilities of their carbohydrate transport systems to uptake specific oligosaccharides are crucial for their oligosaccharide metabolisms. Firstly, the facilitated diffusion system can transport oligosaccharides according to concentration gradients, without requiring additional biological energy. Oligosaccharide/H^+^ and oligosaccharide/Na^+^ transporters allow for the simultaneous transmembrane transport of oligosaccharides and their respective ions. Furthermore, ABC transporters constitute a well-known group of active transport systems, with members such as the Msm EFGK transporter actively participating in bacterial oligosaccharide transport [66]. These ABC transport proteins in bacteria can absorb oligosaccharides with ATP energy input, imparting the ability of oligosaccharides to resist concentration gradients. However, PTS is a prokaryotic-specific transport system. Some members of the PTS, such as pts1BCA, can achieve transmembrane transport and the phosphorylation of oligosaccharides. Among them, phosphoenolpyruvate is both a phosphorylation donor in the bacterial gene library and an energy metabolic pathway for monosaccharide units [67].

### 3.1. Glycosidic Bonds and Monosaccharide Compositions

Different types of glycosidic bonds and monosaccharide compositions affect the digestive resistance of oligosaccharides and further influence their intestinal metabolism and prebiotic properties. For example, compared to β-1,4 low-degree oligosaccharides of lactose, β-1,6-linked low-degree oligosaccharides of lactose exhibit a better growth-stimulating effect on *Bifidobacterium* [68]. Furthermore, low-degree isomaltose, low-degree lactose, and low-degree xylose, when fermented by the same butyric acid bacteria, show different butyric acid production capabilities. Specifically, the butyric acid production of low-degree isomaltose is 1.71 and 2.61 times higher than those of low-degree lactose and low-degree xylose, respectively [69]. The specificity of the glycosidic bonds and monosaccharide compositions of oligosaccharides influences their fermentation characteristics and promotes the production of corresponding enzymes by intestinal bacterial communities. Therefore, the dynamic utilization of oligosaccharides can be adjusted according to the specific requirements of the host [70].

### 3.2. Polymerization

Most oligosaccharides consist of multiple monosaccharides, forming straight or branched chains with different degrees of polymerization, and the chain length of oligosaccharides is a major factor influencing the fermentation of intestinal bacteria. For instance, gut bacteria preferentially ferment short-chain isomaltose (DP = 3) over long-chain isomalto-oligosaccharides (DP = 8). Compared to the intake of long-chain isomaltose, supplementing short-chain isomaltose in the diets of rats can delay the development of colitis by increasing the concentration of butyric acid in cecum contents and the level of glucagon-like peptide-2 in colonic mucosa [71]. When the degree of polymerization is less than seven, *Bifidobacteria* preferentially utilize short-chain human milk oligosaccharides, as short-chain oligosaccharides can be more efficiently captured by *Bifidobacteria*, transported into the cytoplasm, and subsequently fermented [72]. Metabolic experiments with low-degree lactose of different polymerizations revealed that *Lactobacillus rhamnosus* and *Bifidobacterium* mainly consume monosaccharides and disaccharides, while *Lactobacillus acidophilus* consumes trisaccharides [73].

### 3.3. Linear and Branched Structures

The molecular structural characteristics of oligosaccharides are another crucial determinant affecting their breakdown and functionality in the gastrointestinal tract. Oligosaccharides composed of α-glucose with branches have a lower digestion rate in the colon compared to their corresponding linear oligosaccharides. For instance, the digestion rate of linear or branched isomaltose (DP = 2~8) is lower than that of digestible maltodextrin (DP = 2~8), but higher than that of resistant maltodextrin [74]. A study on the functionality of glucoamylase found that maltohexaose can be rapidly hydrolyzed by glucose amylase, compared to branched glucosyl oligosaccharides with a polymerization degree of six. This is because glucose amylase cleaves α-1,4 bonds in linear oligosaccharides at a higher rate than the rate of α-1,4 bonds near α-1,6 bonds in branched oligosaccharides, indicating that the enzyme prefers to cut α-1,4 bonds at the non-reducing end away from the branching point [75]. Therefore, the structural characteristics of oligosaccharides are another determining factor influencing their digestive rate and functional properties in the intestine.

## 4. Application of XG in Intestinal Reactors

### 4.1. XG

**Figure 5:**
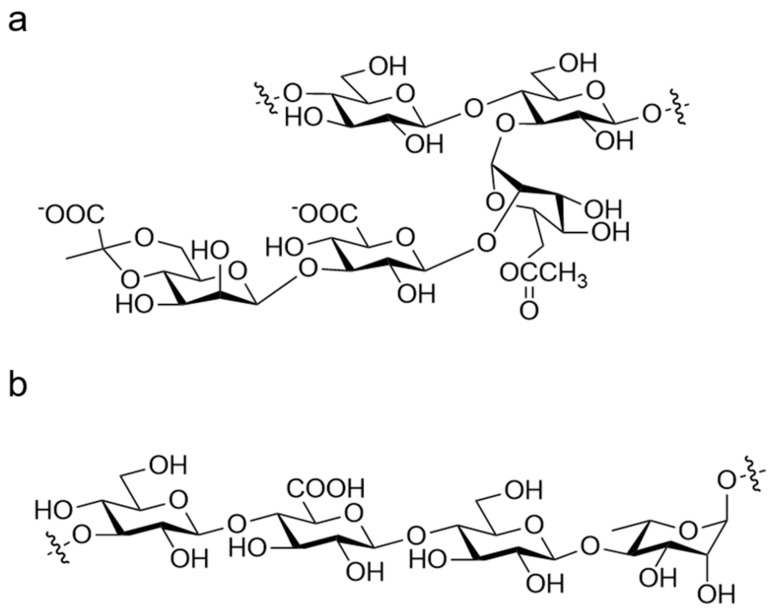
(**a**) Chemical structure of XG; (**b**) chemical structure of GG.

XG is an anionic polysaccharide produced by wild-type *Xanthomonas campestris*, using carbohydrates as the main raw material. Discovered in the early 1960s, it became the second commercially viable microbial polysaccharide [76]. Due to its outstanding functional characteristics, XG remains an important natural microbial extracellular polysaccharide in various industries, including food, pharmaceuticals, and industrial applications. The primary structure of XG reveals it as a high-molecular-weight heteropolysaccharide composed of D-glucose, D-mannose, and D-glucuronic acid in a molar ratio of 2:2:1. The primary structure of XG consists of a glucose backbone linked by β-1,4 glycosidic bonds and side chains composed of trisaccharide units, forming a structural polymer with a “pentasaccharide repeating unit”. The side chains consist of alternating residues of internal α-1,3 mannose, β-1,2 glucuronic acid, and terminal β-1,4 mannose. In the branches, the terminal mannose portion is replaced by pyruvic acid, and the mannose near the main chain undergoes acetylation (Figure 5a). The relative molecular weight of XG typically ranges between 2 × 10^6^ and 5 × 10^7^ Da [77].

Due to its unique molecular structure, XG exhibits outstanding properties, including high viscosity at low concentrations, distinctive rheological characteristics, excellent freeze–thaw stability, and negligible toxicity [78,79]. In 1958, XG was first obtained through microbial fermentation at the Peoria Laboratory. In 1961, the semi-industrial production of XG commenced at CP Kelco in the United States, making it the first company to commercially produce XG in 1963. In 1969, XG was approved by the United States Food and Drug Administration (FDA) as a food additive. From 1971 to 1977, several countries in Europe and America successively approved XG for use as a food additive. In 1975, XG was included in the United States Pharmacopeia (USP) as a pharmaceutical excipient, and in 1980, the FDA designated XG as a safe product. In 1981, the Food Chemical Codex (FCC) proposed specific quality standards for XG as a food additive. In 1983, the Food and Agriculture Organization of the United Nations (FAO) and the World Health Organization (WHO) approved the application of XG in the food industry. In 1986, the FAO and the WHO published quality standards for XG as a food additive [76,80,81]. In specialized medical infant formula, according to the “National Food Safety Standard for Food Additive Use” (GB2760-2014), the maximum allowable usage of XG is 9.0 g/kg [82]. XG imparts unique flavor, viscosity, structure, water-holding capacity, and appearance to food products, while also controlling rheological properties during processing [83]. XG can enhance water retention in baked goods and prolong their shelf life. Additionally, it improves the quality of dough during low-temperature storage before baking, maintaining gas content, smoothness, water-holding capacity, and elasticity throughout repeated processing and kneading, thereby enhancing the structure and volume of gluten-free baked goods [84]. In beverages containing fruit pulp and particles, the thickening and suspension properties of XG create a uniform appearance, providing a certain shape to such beverages while enhancing their quality [85]. The stability and emulsification properties of XG, when used in dairy products like ice cream and cream, help maintain uniformity and stability, reducing the impact of freezing on the flavor and quality of dairy products. Furthermore, the excellent pseudoplasticity of XG enables control over the rheological properties of these dairy products, enhancing their adhesion, quality, and texture, as well as the flavor of flavorings [86,87].

### 4.2. Application of XG in In Vitro Biomimetic Models

XG is the most extensively used microbial edible gum for in vitro digestion experiments, which is attributed to its high food safety and fluid stability. Its main applications are classified into several aspects, which are detailed below:

#### 4.2.1. Fluid Dynamics Evaluation

XG is used to assess fluid dynamics under muscle contraction in in vitro digestion equipment, deepening scientists’ understanding of intestinal peristalsis during the digestive process. Mechanically, muscle contractions generate force, further creating pressure and flow within the cavity. Therefore, in an effective in vitro digestion model, there is a strong desire to simulate biologically relevant fluid dynamic events based on peristaltic patterns. In most in vitro models, the flow profiles have not been well established. Flow visualization techniques, such as positron emission particle tracking (PEPT), particle image velocimetry, and planar laser-induced fluorescence, have been employed to map the flow field. Keppler, S. et al. utilized PEPT to track particles in the in vitro Human Gastrointestinal Tract (IHGS) during simulated meal movements. In a 1% XG solution, the average particle velocity in IHGS was 2.2 mm/s, compared to 220.4 mm/s in water. No chaotic or backflow-like motion was observed using a radiolabeled particle tracer. This may be due to the relatively small effect of the contractile force compared to gravity, leading to a more significant impact on the tracer. This reveals different behavior exhibited by real food particles with lower tracer density [88]. Similarly, Li, Z.T. et al. prepared XG solutions at concentrations of 2.5, 5, 10, 15, 20, and 30 g/L, along with an indicator, for in vitro digestion in the BGR. The study aimed to monitor the mixing process of non-Newtonian fluid-like substances in the reactor. This research revealed the digestion characteristics of XG in the BGR, indicating that XG solutions exhibit shear-thinning properties and good fluid dynamics, as evaluated by the model. They simulated the contraction of stomach contents by mimicking the direct circumferential contraction of the gastric wall. The shear forces generated by the eddies induced by gastric wall contraction increased the kinetic energy in the fluid, reducing viscosity. This implies that the mixing time of non-Newtonian fluids in the BGR is shorter than that of Newtonian fluids [32].

#### 4.2.2. Synbiotics for Auxiliary Colonization

Trombino, S. et al. reported that XG can enhance the release of active substances at the colorectal level, where the pH is close to neutral [89]. Furthermore, due to its susceptibility to colonic enzymes, there is a significant amount of research on its standalone use or combination with other polymers for specific colonic delivery [13]. However, one of the primary challenges of in vitro digestion techniques is replicating the high bacterial cell density and biofilm-associated microorganisms in the intestine, which are crucial for preventing the elimination of less competitive bacteria. To address this issue, the intestinal microbial community is often immobilized in polysaccharide gel beads, starting with a small amount of fecal inoculum, simulating different hosts while maintaining high bacterial diversity and cell density, in addition to facilitating long-term colonization of the intestinal bacterial community in continuous intestinal reactors. Cinquin, C. et al. immobilized bacteria from infant feces through a two-phase dispersion process, fixing them in polysaccharide gel beads (2.5% GG, 0.25% XG) [90]. A single-stage chemostat containing precolonized beads was operated for 52 days, simulating different dilution rates and pH conditions of the proximal, transverse, and distal colon. Nine bacterial groups were retained, with survival rates ranging from 3% to 56%. After one week of fermentation, the beads were well colonized, and each bacterial group maintained stability. Under culture conditions, the growth of immobilized cells, cell release from the beads, and the growth of free cells in the liquid resulted in significant changes in the microbial community. In the proximal phase, the concentration of *Bifidobacterium* spp. increased from 7.4 to 9.6 log CFU/mL; in contrast, during the transverse and distal phases, the concentrations of *Bifidobacterium* spp., *Lactobacillus* spp., and *Clostridium* spp. decreased, and *Staphylococcus* spp. and coliforms concentrations increased. This study represents the first report on the auxiliary roles of GG and XG in the study of intestinal microbiota. The complex microbial community in infant feces can be immobilized in an in vitro continuous colon fermentation model, exhibiting high stability at least at the genus level. The PolyFermS platform, developed by Zihler Berner et.al, whose specific structure was also mentioned earlier (Figure 3), employs a fixation process wherein fecal microbiota is immobilized within a mixed XG–agarose gel bead. This method sustains microbial diversity during prolonged continuous colonic fermentation, achieving a high cell density similar to that in the colon. It enables stable and reproducible cultivation of complex gut communities in multiple reactors, allowing for the simultaneous investigation of various variables, such as environmental parameters, dietary compounds, drugs, and added microorganisms [45]. Building upon this foundation, Pham, V.T., et al. employed the PolyFermS platform, a model that also inoculates fecal microbiota with XG as the immobilization material to simulate the gut microbiota of infants fed with formula milk at the age of 2 months [44]. The fermentation setup included the first inoculum reactor (IR), inoculated with 30% (*v*/*v*) agarose-XG beads immobilizing fecal microbiota, connected to the control reactor (CR) and four treatment reactors (TRs). All TRs and the CR operated in parallel, continuously inoculating 5% fermentation effluent from the IR and supplementing with 95% fresh culture medium, as illustrated in Figure 4. Using the PolyFermS platform, they further investigated the impact of lactic acid metabolism on the composition and activity of gut microbiota, focusing on the effects of pH and retention time (RT) on lactic acid metabolism, with little or no hydrogen production by lactate-utilizing bacteria (LUB). Decreasing the pH from 6.0 to 5.0 increased the quantity of lactate-producing bacteria (LPB), while simultaneously decreasing LUB, leading to the accumulation of lactic acid. At pH 5.0, extending RT from 5 h to 10 h resulted in the complete consumption of lactic acid, accompanied by an increase in LUB. Supplementing DL-lactic acid (60 millimoles) to simulate lactic acid accumulation prompted the production of propionic acid and butyric acid, with no impact on acetic acid production. *Propionibacterium avidum*, a lactate-utilizing bacterium, was able to colonize the reactor within 4 days after inoculation, indicating its ability to compete with other hydrogen-producing lactate-utilizing bacteria.

#### 4.2.3. Probiotic Potential

**Figure 6:**
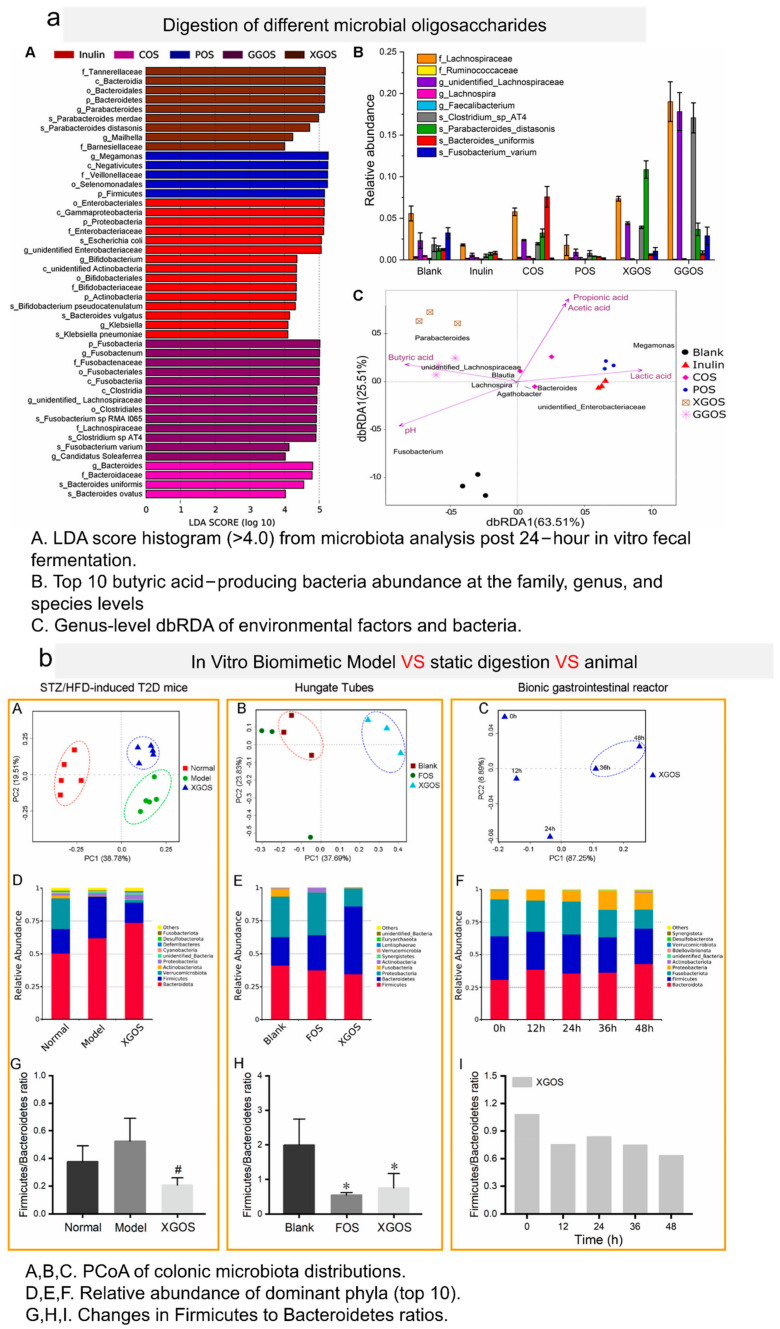
(**a**) Microbiota with significant differences among inulin, COS, POS, XGOS, and GGOS fermentations with fecal bacteria at 24 h in the BGR [6]; (**b**) the effects of XGOSs on colonic microbial communities in T2D mice, Hungate tubes, and the BGR [92]. # *p* < 0.05 compared with the model group in T2D mice; * *p* < 0.05 compared with blank group in Hungate tubes.

Increasing evidence suggests that high-molecular-weight polysaccharides are resistant to digestion by gastrointestinal media, but can be readily digested by the intestinal microbiota [91]. Polysaccharides can be degraded and utilized by the intestinal microbiota in the colon, producing various metabolites such as SCFAs. Moreover, most polysaccharides play a positive role by modulating the composition of the intestinal microbiota. Therefore, investigating the digestion of polysaccharides is particularly important. In vitro digestion measurements capture changes in nutrient contents, digestion and absorption rates, and target component release during the processes of digestion and absorption. Recent studies suggest promising prebiotic applications for the degradation products of XG oligosaccharides (XGOSs). Xu, J. et al. investigated the resistance of thermal gel oligosaccharides, pullulan oligosaccharides, XGOSs, and acacia gum oligosaccharides to salivary and gastrointestinal digestion. Subsequently, they explored their potential as prebiotics through in vitro fermentation using *Lactobacillus* and *Bifidobacterium* and healthy human feces. XGOSs and GG oligosaccharides (GGOSs) exhibit resistance to simulated saliva-based, gastric, and small intestinal digestion, thereby safely reaching the colon. In in vitro fermentation experiments with fecal microbiota from healthy individuals, thermal gel oligosaccharides and pullulan oligosaccharides are predominantly consumed during the 0–12 h fermentation process, leading to a significant increase in concentrations of acetic acid, propionic acid, and butyric acid, along with a decrease in pH. XGOSs and acacia gum oligosaccharides are primarily consumed during the 12–24 h fermentation process, with continuous increases in the concentrations of acetic acid, propionic acid, and butyric acid, along with a decrease in pH during the 0–24 h fermentation process (Figure 6a). Notably, the addition of XGOSs results in the highest production of propionic acid, butyric acid, and total short-chain fatty acids. In in vitro fecal fermentation of XGOSs and acacia gum oligosaccharides, the changes in microbial communities and metabolites are similar, characterized by significant increases in the abundance of butyrate-producing bacteria and butyric acid production. This result may be associated with the shared β(1→4) glycosidic bonds in XGOSs and acacia gum oligosaccharides [6]. Building upon this study, Xu, J. et al. utilized the BGR to investigate the impact of XGOSs on the gut microbiota and their metabolites in healthy individuals and patients with type II diabetes during fecal fermentation [92]. Thin-layer chromatography revealed that XGOSs are primarily degraded during the 24–48 h fermentation process, with a preference for degrading the low-molecular-weight components of XGOSs. In the in vitro dynamic fecal fermentation process of healthy individuals and patients with type II diabetes, compared to the 0–24 h fermentation process, the 24–48 h fermentation process of XGOSs significantly increases the production of acetic acid, propionic acid, and butyric acid, along with the consumption of NaOH, with the most significant increase observed in butyric acid production. In in vitro dynamic fecal fermentation, compared to the fecal microbiota at 0 h and 24 h, XG oligosaccharide fermentation for 48 h significantly alters the gut microbiota structure in both healthy individuals and patients with type II diabetes. This includes a consistent reduction in the *Firmicutes*/*Bacteroidetes* ratio and an increase in the relative abundance of *ParaBacteroides*, *Hungatella*, and UBA1819 genera in the fecal microbiota of both healthy individuals and patients with type II diabetes. In the in vitro dynamic fecal fermentation of XGOSs, the enrichment of butyrate-producing bacteria may be associated with a significant increase in butyric acid production (Figure 6b). Fangwei Liu et al. examined the digestion of different biscuits containing konjac glucomannan, arabinogalactan, or XG in the gastrointestinal tract. In the group with added XG, the fecal microbiota of healthy subjects exhibited the greatest reduction in pH (2.13 ± 0.02) during the fermentation process, and the carbohydrate utilization rate was higher than in the groups with other biscuits [93]. In the group with added XG in biscuits, there were increases in the relative abundance of *Coprococcus*, *Ruminococcus*, and *Roseburia*. The *Ruminococcaceae* uncultured genus 13 (R. UCG13) from the group possesses a complete enzyme library, capable of utilizing GH5 enzymes to hydrolyze natural XG, producing pentasaccharides. The released pentasaccharides can be consumed by R. UCG13 itself and intestinal *Bacteroidetes*. *Ruminococcus* and *Roseburia* are major butyrate-producing bacteria, and they are associated with the degradation of polysaccharides and fibers. The increased relative abundance of *Coprococcus* is negatively correlated with the development of diabetes.

Recent studies have indicated diverse variations in the structure and properties of XG across different digestion models, encompassing its interactions with other components, its stability during the digestion process, and its impact on the digestion rates of nutritional substances. Some of these studies are listed in Table 1, and these research findings are crucial for comprehending the behavior of XG in the human digestive system and its applications in the food industry and pharmaceutical field.

## 5. Application of GG in Intestinal Reactors

### 5.1. GG

GG is a linear anionic polysaccharide produced by Sphingomonas Elodea. It was approved by the U.S. Food and Drug Administration in 1992 and became the third microorganism-derived extracellular polysaccharide authorized for use in food. Its backbone structure is a typical linear molecule, consisting of repeating units of →3)-β-D-glucose-(1→4)-β-D-glucuronic acid-(1→4)-β-D-glucose-(1→4)-α-L-rhamnose-(1→(Figure 5b). Its molecular weight is approximately 5 × 10^5^~1 × 10^6^ Da [102]. The spatial structure of GG is formed by the aggregation of double helical structures, influenced by physical forces, hydrogen bonds, and van der Waals forces. Its double helical structure exhibits cohesion, and under the secondary interaction of hydrogen bonds, it further cross-links to form a three-dimensional macromolecular network structure. Studies have shown that GG undergoes a thermally reversible process of transitioning from a helical to a double helical structure during gelation. As the temperature decreases, it transitions from a disordered single-chain state to an ordered double helical structure [103,104]. Due to its unique structure, GG exhibits outstanding properties such as stability, gelling ability, and thickening capacity. Additionally, it possesses good safety and solubility, making it widely used in various fields, including food, biopharmaceuticals, and agricultural environments. In the biomedical field, GG serves as a drug delivery vehicle and can also be used in cartilage tissue regeneration [105,106]. In the agricultural sector, GG composite hydrogels, due to their high water retention capacity, can release fertilizers in a controlled manner [107]. Although there has been research on applying GG to wound dressings, its application effectiveness is less than ideal compared to other bacterial polymers, due to its soft texture and poor thermal stability. However, with the advancement of 3D printing technology, this issue has been partially addressed. Some characteristics of GG, such as its cross-linking potential, mediated by extremely low cation concentrations, excellent gel formation ability at 37 °C, enhanced monodispersity, low immunogenicity, and outstanding rheological properties, make it an ideal choice for bio-printing inks [108,109]. In 1988, following a series of toxicology tests, Japan approved the use of GG in food. Subsequently, the United States (1990) and Europe (1995) also approved the use of GG. According to reports, the consumption of GG has no adverse effects. In the food industry, GG can form a network structure at the interface of continuous phase and oil–water, serving as a stabilizer for emulsion gels, including jams, jellies, water-based gels, pie fillings, puddings, processed foods, sugar coatings, sugar glazes, and dairy products (ice cream, yogurt, milkshakes, and gelled milk). Additionally, GG can be used to replace unnecessary ingredients, such as fat, to manufacture products that induce a feeling of fullness or low-calorie diets [110].

### 5.2. Application of GG in In Vitro Digestion

In applications, GG is similar to XG, and they are often used alternately or in combination to assess the crushing ability, fluid dynamics, and probiotic colonization ability of bioreactors. Due to its ability to form strong gels, GG is used extensively in the preparation of microcapsules. GG can be classified into high-acylated (HAG) and low-acylated (LAG) types. Natural GG is a high-acylated GG, which, after KOH saponification, removes acyl and glycerol groups, forming low-acylated GG with excellent gel properties. The gelation process of high-acylated GG is temperature-sensitive, typically forming a gel at around 70 °C. Due to the acyl groups, the gel formed by high-acylated GG exhibits characteristics such as adhesiveness, high elastic modulus, and softness. In contrast, the gel formation of low-acylated GG relies on the control of cations and temperature, with gelation occurring at temperatures between 30 and 40 °C. In aqueous solutions, the gelation of low-acylated GG is mainly achieved through the right-hand rotation aggregation of GG molecules via intermolecular hydrogen bonding, as well as the electrostatic interaction between carboxyl groups and cations, leading to the aggregation of the right-hand rotation structure into a three-dimensional network structure, thereby trapping water molecules to form a gel. Since low-acylated GG removes acyl groups, its spatial hindrance is significantly reduced, endowing the gel with high strength and hardness. Microcapsules encapsulating probiotics made from GG have been applied in in vitro digestion experiments. Sun, W. et al. used GG–guar gum to immobilize bifidobacteria, and the study found that the tolerance of probiotics to high-acid environments significantly increased after encapsulation, and the storage stability in yogurt also improved significantly [111]. Nag, A. et al. reduced the pH using glucono-delta-lactone (GDL), causing the gelation of a GG and sodium caseinate mixture, resulting in the uniform particle size distribution of encapsulated *Lactobacillus*, and, after simulated gastric and bile salt treatments for 30 min, the survival rate of *Lactobacillus* in dry cheese significantly increased (*p* < 0.001), surpassing that of free cells [112]. Dixit et al. evaluated GG–chitosan polyelectrolyte complexes in bead form to prolong the potential for drug-specific release in the gastrointestinal tract. These composite beads exhibited excellent buoyancy in the acidic pH environments of gastric fluid and better control over the release of the drug lufoprazine [113]. Rosas-Flores, W. et al. prepared microcapsules using GG and alginates, embedding *Lactobacillus delbrueckii* and *Lactobacillus helveticus* through internal ionic gelation technology. The study indicated that, at a stirring rate of 400 rpm, the encapsulation efficiency of probiotic microcapsules was the highest [114]. In research on prebiotics, Gao, M. et al. prepared GGOSs with different degrees of polymerization through acid and enzymatic hydrolysis. They conducted product analysis and identification of the GGOSs obtained by the two degradation methods, analyzing structural differences. Based on this, they performed in vitro simulated digestion experiments on GGOSs, analyzing their degradation in the upper digestive tract. Finally, different degrees of GGOSs were subjected to in vitro fecal fermentation to explore the effects on intestinal microbial community structure and metabolite differences. This is the first study on GGOSs as prebiotics in an in vitro digestion dynamic model, with specific procedures as follows: First, GGOSs with a wide range of degrees of polymerization were prepared by acid hydrolysis. The optimized conditions included a substrate concentration of 5 g/L, a hydrochloric acid concentration of 0.5 mol/L, and a hydrolysis time of 3 h, yielding GGOSs with predominant degrees of polymerization, ranging from 2 to 8. Subsequently, GGOSs were prepared using a one-step method, coupling Baker’s yeast with a polysaccharide addition. The optimized fermentation degradation conditions were an initial GG addition of 2 g/L, fermentation for 96 h, and a methanol addition of 1%, resulting in GGOSs with a single degree of polymerization. Structural analysis of the GGOSs obtained by the two degradation methods was then performed. Infrared spectroscopy analysis indicated no structural changes in the GGOSs obtained by acid hydrolysis, while those obtained by enzymatic hydrolysis showed the presence of C = C structures. Monosaccharide composition analysis results showed that the monosaccharide composition of acid-hydrolyzed GGOSs was consistent with GG, while enzymatically hydrolyzed GGOSs consisted of glucose and xylose. Mass spectrometry results showed that acid-hydrolyzed GGOSs, after separation by gel filtration chromatography, yielded components with degrees of polymerization including 3 and 4 (GOS1) and components with degrees of polymerization including 6 and 8 (GOS2), while enzymatically hydrolyzed GGOSs only contained components with a degree of polymerization of 4 (GOS3). GGOSs resisted digestion in the upper digestive tract and successfully reached the colon. In vitro simulated digestion experiments showed, through thin-layer chromatography, ion chromatography, and reducing sugar content determination, that GGOSs had no free monosaccharides during in vitro simulated digestion, maintaining their degree of polymerization without a significant increase in reducing sugar content. Oligosaccharides with different degrees of polymerization exhibited different capabilities for enriching various intestinal bacterial groups, and the degree of polymerization showed a negative correlation with the fermentation rate. After in vitro fecal fermentation, it was found that GOS1 had a stronger enrichment ability for genera such as *Clostridium*, *Faecalibacterium*, and *Selenomonas*, GOS2 had a stronger enrichment ability for genera such as *Bacteroides* and *Ruminococcus*, and GOS3 had a stronger enrichment ability for genera such as *Megamonas*, *Lachnospiraceae*, and *Oscillospiraceae*. Short-chain fatty acids produced by intestinal bacteria after in vitro fecal fermentation were measured, and the results showed that the production activities of acetic acid, propionic acid, and butyric acid by GOS1, GOS2, and GOS3 were higher than that in the negative control group, with higher production of short-chain fatty acids by GOS1 and GOS3 compared to GOS2 [115]. Recently reported applications of GG in static digestion models have revealed various interesting findings, with the main research outcomes summarized in Table 2. For instance, GG encapsulating anthocyanins effectively retains them in the stomach and releases them in the intestines without inhibiting the proliferation of intestinal epithelial cells. Some physically cross-linked GG microcapsules maintain their shape after gastric digestion and disintegrate during intestinal digestion, making them suitable for intestinal delivery systems. Additionally, guava with added GG exhibits higher elasticity in temperature scans, and its total antioxidant activity and average extractable polyphenol values closely resemble those of guava pulp, indicating an enhancement in antioxidant properties with the addition of GG. Based on acylation differences in GG, high-acyl GG as an emulsifier in β-carotene emulsions exhibits stability during oral and gastric digestion and enhances the bioavailability of β-carotene during simulated intestinal digestion. These research findings provide crucial references and guidance for the further development of novel foods and drugs with the potential to treat and prevent intestinal diseases.

## 6. Conclusions and Future Trends

The study of the impact of microbial gums on the gut microbiota has evolved from compositional analysis to functional exploration. In vitro digestion has provided a new perspective for investigating the probiotic activity of microbial gums. As our understanding deepens regarding the digestion and fermentation characteristics of complex food components and gut microbial communities, research on the edible microbial gum family will continue to evolve. The structures, binding capacities, transport barrier characteristics, and digestive functions of microbial gums are closely related. While diets rich in microbial gums offer a forward-looking nutritional intervention strategy for improving gut health, the multifactorial nature of their compositions, interactions, processing-induced changes, and physiological responses poses challenges to a dietary structure rich in microbial gums. Further research is needed to address the following issues:

- Multi-faceted exploration of interactions: a diverse range of microbial gums and various digestion methods (such as static digestion and in vitro dynamic digestion) should be employed to comprehensively explore the interactions among diet, host health, and metabolic regulation.
- Innovations in preparation methods and expanded applications: new preparation methods for microbial gums as prebiotics need to be developed, and novel applications as synbiotics also warrant exploration.
- Detailed mechanistic insights: extensive research is required to elucidate the detailed mechanisms underlying the relationship between microbial gum structure and host health, mediated by the gut microbiota.
- Personalized nutrition with microbial polysaccharides: the development and application of personalized nutritional microbial gums, based on the functional characteristics of the gut microbiota.
- Structural, functional, and environmental associations: given that microbial gums vary with bacterial sources and processing history, attention should be paid to the associations of these variations with structure and activity. These factors often co-determine the metabolic pathways and cross-feeding interactions within the gut microbial community, ultimately influencing host health.
- More realistic models for digestion detection and evaluation: in vitro experiments are constrained by model simplifications and experimental conditions, and they are unable to fully capture the diversity and dynamics of food in the actual intestines. Continuous updates and iterations are essential to overcome the limitations of in vitro digestion. Researchers must exercise caution in interpreting in vitro experiment results at this stage, combining them with other research methods for a comprehensive understanding.

In future studies, it is recommended to utilize microbial gum interventions for precise modulation of the microbiota, aiming to maximize health benefits. Providing detailed dosage–effect information and establishing relevant databases will be crucial in interpreting the impact of microbial gums on the relationship among microbial gums, gut microbiota, and human health.

## Figures and Tables

**Table 1:** Structural and property variations of XG in different digestion models.

**Sample**	**In Vitro Digestion Model**	**Changes During Digestion**	**Key Findings**	**Reference**
XG emulsion with/without Tween 80	Three-step in vitro digestion model	Fat coagulation; increased viscoelasticity, due to weak points in XG structure; lower fat digestion rate in XG emulsion with Tween 80	Tween 80-containing XG emulsion resists fat digestion and reduces FFA levels. XG solution shows decreased viscoelasticity and more flexible chains. Increased gastric viscoelasticity in XG emulsion, due to weak points in structure.	[94]
XG–palm oil system	Three-step in vitro digestion model	Decomposition of XG–palm oil system; structural changes; formation of metabolites	After gastric cultivation: increased extrusion force, modulus of elasticity, and viscosity in XG system; associated with higher water dilution and increased fat droplet size.	[95]
XG	Infogest COST Action	Aggregation, maintaining structure and viscosity during gastric stage	XG exhibits aggregation during digestion, maintaining its structure and viscosity during the gastric stage.	[96]
Kudzu starch and XG	Static digestion	Changes in molecular structure; formation of degradation products	Increasing XG concentration enhances interaction with kudzu starch, slowing down its hydrolysis during in vitro digestion. XG oligosaccharides may influence molecular structure.	[97]
XG	Infogest simulated digestion model	Highest viscosity of XG at the end of digestion, followed by GG and LBG, at low and high viscosities	XG significantly reduces glucose and free amino acid release at high viscosity; linear relationship with protein and starch digestion.	[98]
Whey Protein Isolate (WPI) and XG	Static digestion	Changes in emulsion particle size; degradation of XG; interaction between WPI and XG	WPI-XG stable emulsion affects digestion process of emulsion particles, resulting in lower digestion rates of fat and carotenoids.	[99]
Peptides–carrageenan–XG	Static digestion	Changes in molecular structure; formation of degradation products	ABTS ion, hydroxyl radical, and iron ion clearance rates were 62.83%, 74.81%, and 0.035 mg/mL, respectively.	[100]
XG	Static digestion	Changes in molecular structure (FTIR and NMR measurements)	After 2 h of exposure in SGF, changes in the molecular structure of XG were observed.	[101]

**Table 2:** Key outcomes from recent GG applications in static digestion models.

**Sample**	**Changes During Digestion**	**Functional Evaluation**	**Key Findings**	**Reference**
Anthocyanins encapsulated in GG	Anthocyanins retained in GG system in the stomach, released in the intestine.	Biocompatibility test with IEC-6 cells; GG system does not inhibit cell proliferation	GG-encapsulated anthocyanins effectively retained in the stomach and safely released in the intestine, showing potential as a safe and effective approach for treating and preventing intestinal diseases.	[116]
GG Microcapsules by physical cross-linking	Assessment of microcapsule size distribution, morphology, and Zeta potential before and after in vitro digestion	Evaluation of long-term stability	Microcapsules maintain shape after gastric digestion, disintegrate during intestinal digestion, suitable for intestinal delivery systems. Chitosan coating slows capsule disintegration in intestinal digestion, indicating coating aids in control of release characteristics.	[117]
Mixture of GG and Guava	Guava with added GG exhibits higher elasticity during time scan.	Evaluation of long-term stability, ascorbic acid, total antioxidant activity, and polyphenol content	Guava with added GG shows total antioxidant activity and average total extractable polyphenols close to values of guava pulp.	[118]
β-carotene Emulsion with High-acyl GG as emulsifier	Average particle size (MPS); emulsion yield (EY); dynamic stability	Bioavailability of β-carotene	Stable during oral and gastric digestion phases, MPS and ZP changes between 2.5 μm and 3.0 mV. In simulated intestinal digestion, β-carotene releases forms micelles; HA-β-carotene emulsion enhances release rate of FFA, improving β-carotene bioavailability.	[119]
Hydrogel beads comprising GG and Resistant Starch	Changes in molecular structure; introduction of carboxyl groups	pH sensitivity; drug loading efficiency	Gel beads exhibit good stability in simulated gastric fluid and continuous release of resveratrol in simulated intestinal fluid.	[120]
Mixture of Glycerol Monostearate–Beeswax Oleogel and High-acyl GG Hydrogel	Changes in molecular structure; rheological studies	Mechanical strength (storage modulus, hardness) increases with increasing oleogel content	Colloidal structure exhibits oil gel–water gel configuration; increasing oleogel content results in larger droplets. Rheological results indicate all colloids exhibit solid characteristics as storage modulus exceeds loss modulus.	[121]

## Data Availability

No new data were created or analyzed in this study. Data sharing is not applicable to this article.

## Supplementary Materials

The following supporting information can be downloaded at: https://www.mdpi.com/article/10.3390/foods13050713/s1.
