# Supplementary material for: New Horizons in Probiotics: Unraveling the Potential of Edible Microbial Polysaccharides through In Vitro Digestion Models
Source: Foods. 2024 Feb 26;13(5):713. doi: 10.3390/foods13050713 (PMC10931329; doi:10.3390/foods13050713)
Supplement: Supplementary file 1 [file foods-13-00713-s001.zip › foods-2861734-supplementary.pdf]

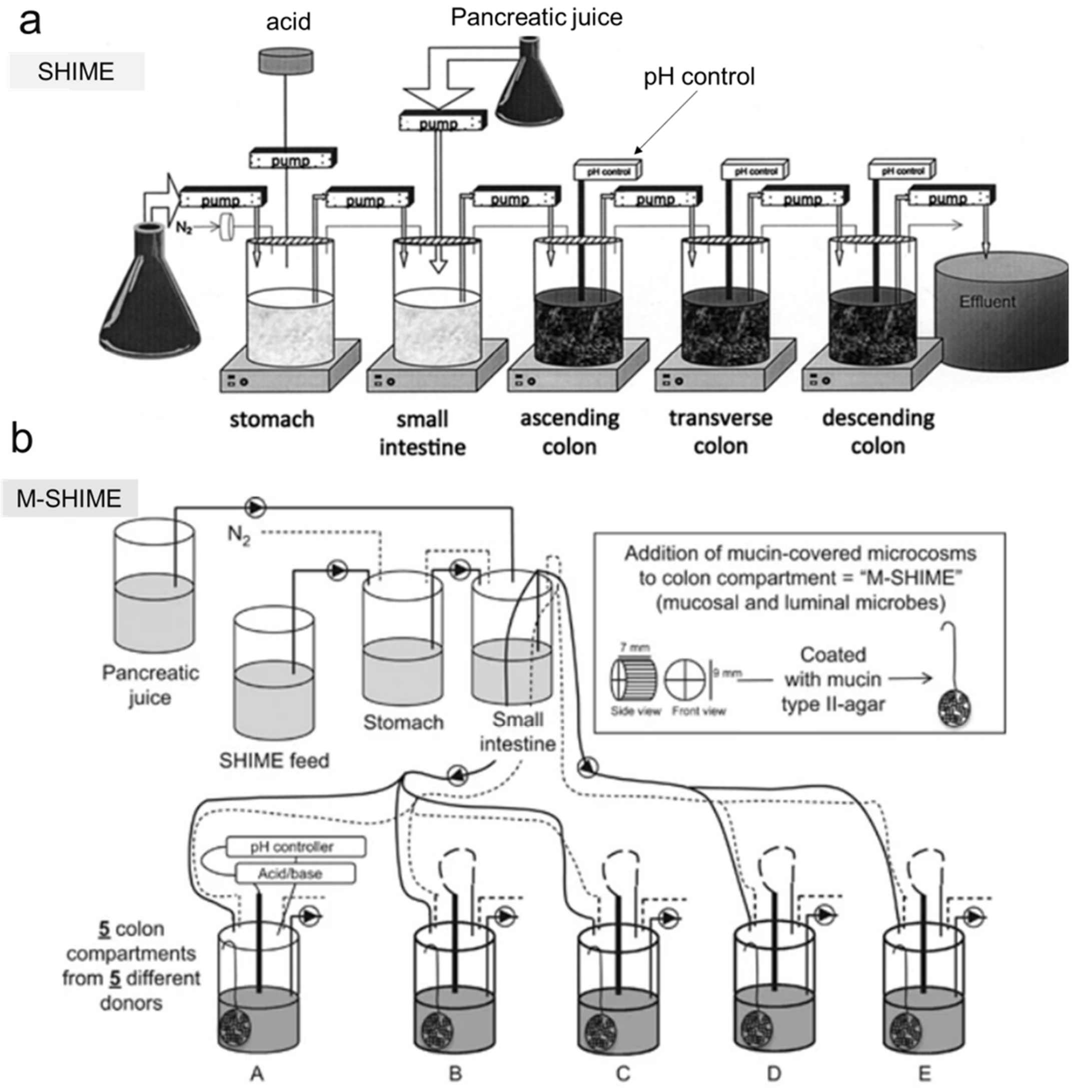

Supplementary Figure.S1: (a) Schematic representation of SHINE [33]; (b) Enhanced schematic representation of M-SHINE, which builds upon SHINE by incorporating mucin-covered microorganisms in the colonic compartments for a more authentic physiological biomimicry [36].

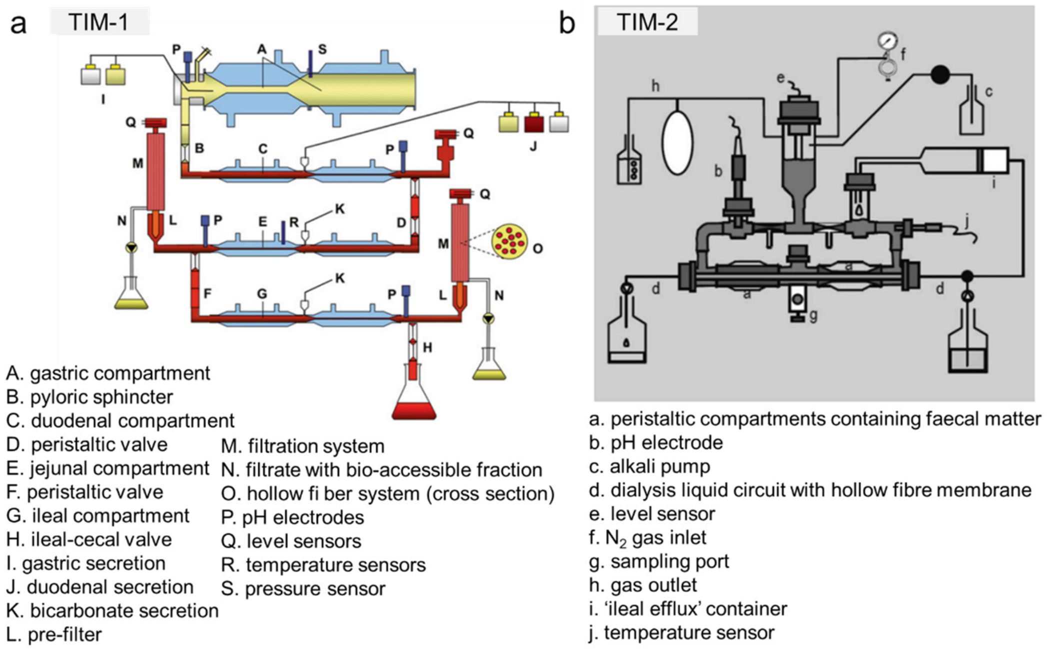

Supplementary Figure.S2: (a) Schematic representation of TIM-1 [30] ;(b) Schematic representation of TIM-2 [29].
